# Effects of carbamazepine on semen parameters in men with newly diagnosed epilepsy

**Published:** 2015-07-06

**Authors:** Ali Asadi-Pooya, Mohsen Farazdaghi, Nahid Ashjazadeh

**Affiliations:** 1 Department of Neurology, School Medicine, Shiraz University of Medical Sciences, Shiraz, Iran AND Department of Neurology, Sidney Kimmel Medical College AND Jefferson Comprehensive Epilepsy Center, Thomas Jefferson University, Philadelphia, PA; 2 Department of Neurology, School of Medicine, Shiraz University of Medical Sciences, Shiraz, Iran

**Keywords:** Carbamazepine, Epilepsy, Semen, Men

## Abstract

**Background:** We investigated the effects of carbamazepine (CBZ) on semen parameters in men with newly diagnosed epilepsy, by performing semen analysis before starting any antiepileptic drugs, and then after starting CBZ, to determine the role and effects of CBZ in creating abnormalities in sperm analysis in these patients.

**Methods: **In this prospective study, eight male patients 20-40 years of age who were referred to the outpatient epilepsy clinic at Shiraz University of Medical Sciences, Iran, from 2009 to 2012, due to new-onset seizure(s) were studied. A semen analysis was performed. CBZ was started and after at least 3 months of taking CBZ, another semen analysis was requested to determine the changes in semen quality. Statistical analyses were performed using non-parametric Wilcoxon test.

**Results: **Mean age of the patients was 28.5 ± 3.5 years. 7 (87.5%) patients had temporal lobe epilepsy and 1 (12.5%) had parietal lobe epilepsy. The mean follow-up period was 5.5 ± 0.9 months. We observed that semen quality (concentration, progressive motility, morphology) has significantly changed in patients with newly-diagnosed epilepsy after being treated with CBZ (P = 0.012 for all indices).

**Conclusion:** This study shows the direct effects of CBZ in causing changes in semen quality in men with epilepsy. Abnormalities in sperm concentration, morphology and motility, which were observed in the current study, might play a significant role in causing reduced fertility in men with epilepsy.

## Introduction

Reproductive disorders are more common among men with epilepsy than in the general population.^1^ Both epilepsy and antiepileptic drugs (AEDs) may play a role in creating these problems, however, the underlying mechanisms have not yet been identified clearly and separating the direct effects of epilepsy versus AEDs has always been difficult.^2^ Infertility, morphological changes in testes and abnormalities in sperm analysis have been reported in patients taking sodium valproate.^3^^-^^5^ Carbamazepine (CBZ) had negative effects on sperm analysis in both animal and human studies.^6^^-^^8^

In this study, we investigated the effects of CBZ on semen parameters in men with newly diagnosed epilepsy, by performing semen analysis before starting any AEDs, and then after starting CBZ, to determine the role and effects of CBZ in creating abnormalities in sperm analysis in these patients.

## Materials and Methods

In this prospective study, eight male patients, who were referred to the outpatient epilepsy clinic at Shiraz University of Medical Sciences, Iran, from January 2009 to January 2012, due to new-onset seizure(s) were studied. Inclusion criteria were patients aged 20-40 years at the time of referral; whose seizures were considered to be epileptic in nature [based on the clinical grounds and the electroencephalographic (EEG) and/or imaging studies]; who has not used even a single dose of any AED before their first visit; and finally, has not had any other medical or psychological disorders requiring long-term treatment in their life before. Out-patient EEG and brain magnetic resonance imaging studies were performed in all patients at the time of referral. After determining the nature of their epilepsy and when we considered that CBZ is an appropriate drug for their condition, we discussed the study procedures and purposes with the patients. When they signed the informed consent forms, a semen analysis was requested. All patients were instructed how to collect the samples. Sperm analysis was done by sperm quality analyzer IIC-P (Medical Electronic Systems, Los Angeles, CA). CBZ was started (after semen analysis was done) with 200 mg per day and titrated to 400-600 mg per day in 1-2 weeks. Our strategy was to prescribe CBZ 400 mg per day in patients with a single seizure, who were willing to take medicine, and administer CBZ 600 mg per day in patients who had more than one seizure before their referral. After at least 3 months of taking CBZ, another semen analysis was requested and performed at the same laboratory. All patients tolerated CBZ without any significant side-effects.

Statistical analyses were performed using non-parametric Wilcoxon test to determine potentially significant differences before and after taking CBZ. A P < 0.050 was considered significant. This study was conducted with approval by Shiraz University of Medical Sciences Review Board.

## Results

Eight patients were studied. Mean age of the patients was 28.5 ± 3.5 years. 7 (87.5%) patients had temporal lobe epilepsy and 1 (12.5%) had parietal lobe epilepsy. The mean follow-up period was 5.5 ± 0.9 months. The results of the semen analyses of the patients before and after CBZ therapy are summarized in table 1. 

## Discussion

Reproductive disorders are common among men with epilepsy.^9^ The etiology of reproductive and sexual dysfunction in men with epilepsy has been attributed to a number of possible etiologies; including psychosocial stress, AEDs, and epilepsy itself.^9^ Separating the direct effects of epilepsy versus AEDs have always been difficult. Role of AEDs in sexual dysfunction among patients with epilepsy has been investigated repeatedly. It has been speculated that AEDs can induce various hormonal abnormalities; in particular, the use of the liver enzyme inducing AEDs, such as phenytoin and CBZ, which increases serum sex hormone binding globulin concentrations. This increase leads to diminished bioactivity of testosterone, which may result in diminished potency and thus reduced fertility.^10^ In a number of studies, it has been reported that men with epilepsy treated with CBZ, had altered semen quality compared with healthy controls.^7^^,^^8^ However, no human study has ever investigated the semen quality in patients with epilepsy, before and after treatment with any AEDs. In the current study, we observed that semen quality has significantly changed in patients with newly-diagnosed epilepsy after being treated with CBZ. This shows the direct effects of CBZ in causing changes in semen quality in men with epilepsy. Abnormalities in sperm concentration, morphology and motility, which were observed in the current study, might play a significant role in causing reduced fertility in men with epilepsy.^7^ Our findings are concordant with the observation of reduced fertility among male patients with epilepsy reported in previous studies.^10^ Further, larger studies with CBZ and other AEDs, particularly new AEDs, are necessary to determine the role of each AED in causing reproductive disorders among men and women with epilepsy.

## Conclusion

This study shows the direct effects of carbamazepine in causing changes in semen quality in men with epilepsy. Abnormalities in sperm concentration, morphology, and motility, which were observed in the current study, might play a significant role in reduced fertility in men with epilepsy.

***Limitation***

The main limitation of this study is the small number of the patients enrolled in the investigation.

**Table 1:** Semen parameters before and after taking carbamazepine (CBZ) in men with newly-diagnosed epilepsy

**Semen parameters**	**Before starting ** **CBZ (mean ± SD)**	**While taking CBZ ** **(mean ± SD)**	**Percentage change ** **between means (%)**	**P **
Mean volume (ml)	3.25 ± 1.46	3.50 ± 1.22	7.70	0.672
Mean concentration (million/ml)	78.37 ± 25.99	54.50 ± 32.36	−30.45	0.012
Mean progressive motility (%)	50.75 ± 9.25	41.75 ± 14.50	−17.73	
Mean normal morphology (%)	35.00 ± 6.80	28.62 ± 9.27	−18.23	
Mean motile sperm count (million/ml)	42.65 ± 20.75	26.66 ± 21.88	−37.49	
Mean functional sperm count (million/ml)	26.54 ± 16.99	15.48 ± 15.63	−41.67	
Mean sperm motility index	214.25 ± 70.82	152.12 ± 85.91	−28.99	

CBZ: Carbamazepine; SD: Standard deviation

## Notes:

**How to cite this article: **Asadi-Pooya A, Farazdaghi M, Ashjazadeh N. Effects of carbamazepine on semen parameters in men with newly diagnosed epilepsy. Iran J Neurol 2015; 14(3): 168-70.

## References

[1] Chen SS, Shen MR, Chen TJ, Lai SL (1992). Effects of antiepileptic drugs on sperm motility of normal controls and epileptic patients with long-term therapy. Epilepsia.

[2] Gates JR (2004). Epilepsy versus antiepileptic drugs and gonadal function in men. Neurology.

[3] Curtis VL, Oelberg DG, Willmore LJ (1994). Infertility secondary to valproate. Journal of Epilepsy.

[4] Sveberg RL, Tauboll E, Berner A, Berg KA, Aleksandersen M, Gjerstad L (2001). Morphological changes in the testis after long-term valproate treatment in male Wistar rats. Seizure.

[5] Yerby MS, McCoy GB (1999). Male infertility: possible association with valproate exposure. Epilepsia.

[6] Soliman GA, Abla A, el M (1999). Effects of antiepileptic drugs carbamazepine and sodium valproate on fertility of male rats. Dtsch Tierarztl Wochenschr.

[7] Roste LS, Tauboll E, Haugen TB, Bjornenak T, Saetre ER, Gjerstad L (2003). Alterations in semen parameters in men with epilepsy treated with valproate or carbamazepine monotherapy. Eur J Neurol.

[8] Reis RM, de Angelo AG, Sakamoto AC, Ferriani RA, Lara LA (2013). Altered sexual and reproductive functions in epileptic men taking carbamazepine. J Sex Med.

[9] Herzog AG (2008). Disorders of reproduction in patients with epilepsy: primary neurological mechanisms. Seizure.

[10] Verrotti A, Loiacono G, Laus M, Coppola G, Chiarelli F, Tiboni GM (2011). Hormonal and reproductive disturbances in epileptic male patients: emerging issues. Reprod Toxicol.

